# A Rare Case of Acetaminophen Toxicity Leading to Severe Kidney Injury

**DOI:** 10.7759/cureus.5003

**Published:** 2019-06-25

**Authors:** Maryam Saleem, Hassaan Iftikhar

**Affiliations:** 1 Internal Medicine, Waterbury Hospital, Waterbury, USA; 2 Internal Medicine, St. Francis Medical Center, Seton Hall University, Trenton, USA

**Keywords:** acetaminophen, acute kidney injury, hemodialysis, n-acetyl cysteine (nac)

## Abstract

Acetaminophen is one of the most common analgesic medications available over the counter. Acetaminophen overdose can cause both hepatic and renal injuries. The literature suggests the incidence of acute kidney injury is around 2% - 10% in those with acetaminophen overdose. We report a case of acute kidney injury from acetaminophen overdose requiring hemodialysis.

## Introduction

Acetaminophen (acetyl-para-aminophenol or APAP) is well-known for its hepatotoxicity when taken in an excessive amount. However, it can be equally harmful to the kidneys, giving rise to acute kidney injury (AKI) when ingested in toxic amounts. AKI occurs in about 2% - 10% of patients with APAP overdose and the rise in serum creatinine is usually seen two to five days after ingestion [1]. Despite this, APAP overdose requiring hemodialysis is relatively rare with documented examples described in a few case reports [2-5]. It is important to distinguish APAP-induced renal failure from other causes of kidney damage, including acute tubular necrosis from hypovolemia, along with hepatorenal syndrome, which can also happen in APAP overdose.

## Case presentation

A 39-year-old male with a history of schizoaffective disorder, bipolar disorder, and multiple prior suicide attempts presented with 10 hours of severe abdominal pain after ingesting over 100 gms of APAP two days earlier. On the morning of admission, the patient felt diffuse, severe abdominal pain, most prominent in the right upper quadrant, associated with nausea and vomiting. Upon evaluation in the emergency room, the patient was afebrile with a blood pressure of 110/66 mmHg, a heart rate of 100 beats per minute (BPM), and a respiratory rate of 16 with oxygen saturation of 99% on room air. His physical exam was significant for diffuse tenderness to palpation in right upper quadrant and epigastric area. The patient’s complete blood count was within normal limits. Other initial laboratory workup at the time of admission is shown in Table 1.

**Table 1:** Initial Laboratory Workup After Acetaminophen Overdose

Laboratory test	Results
Aspartate aminotransferase (AST)	890 units/L (Normal: 10 - 40 units/L)
Alanine aminotransferase (ALT)	651 units/L (Normal: 7 - 56 units/L)
Alkaline phosphatase (ALP)	112 units/L (Normal: 40 - 120 units/L)
Total protein	7.5 g/dL (Normal: 6 - 8.3 g/dL)
Albumin	4.4 g/dL (Normal: 3.2 - 5.1 g/dL)
Acetaminophen level	< 10 mcg/mL

The patient was admitted due to transaminitis secondary to APAP toxicity and started on intravenous N-acetyl cysteine (NAC) protocol for 16 hours. The initial plan was to trend liver functions tests, international normalized ratio (INR), and APAP levels every six to eight hours and two hours prior to a 16-hour infusion being completed to determine if NAC was still needed. Table 2 describes his trend of liver function tests and APAP levels.

**Table 2:** Trend of Liver Function Tests and Acetaminophen Levels ALP: alkaline phosphatase; ALT: alanine aminotransferase; AST: aspartate aminotransferase; INR: international normalized ratio; N: normal

Laboratory workup	Day 1	Day 2	Day 3	Day 4	Day 5	Day 6
Acetaminophen (N: < 10 mcg/mL)	< 10	74	< 10	< 10	< 10	< 10
ALT (N: 10 - 40 units/L)	898	2,842	> 7,500	7,154	5,900	3,400
AST (N: 7 - 56 units/L)	701	2,066	> 7,500	1,481	930	217
ALP (N: 40 - 130 units/L)	114	98	78	92	84	116
Total protein (N: 6 - 8.3 g/dL)	7.5	6.2	5.3	4.5	4.4	4.2
Albumin (N: 3.2 - 5.1 g/dL)	4.4	3.5	2.9	2.3	2.2	2.1
INR	1.3	5.0	2.7	2.4	1.8	1.4

NAC was discontinued on Day 6 of hospitalization as the AST and ALT were less than < 3,500 units/L and APAP levels remained normal. However, despite normalization of liver function tests, his blood urea nitrogen (BUN) and creatinine trended up daily (data can be found in Table 3).

**Table 3:** Trend of BUN and Creatinine BUN: blood urea nitrogen

Renal Function Tests	Day 1	Day 2	Day 3	Day 4	Day 5
Creatinine (mg/dl)	0.6	0.6	2.38	5.1	5.7
BUN (mg/dl)	18	12	28	38	40

A nephrology consult was placed on Day 3 in the setting of the patient's rising creatinine. His urinalysis was negative for blood, protein, and white blood cells. Urine pH and specific gravity were also normal. Urine sodium was 34 mmol/L (normal: 30 - 90 mmol/L). Ultrasound of the retroperitoneum was negative for any evidence of obstruction. Initial differentials were initially acute tubular necrosis (ATN) from hypovolemia, acute tubular injury from APAP, or hepatorenal syndrome. We considered ATN, but there was no evidence of hypovolemia or poor perfusion as evidenced by a negative lactic acid and normal blood pressure, the absence of antihypertensives or diuretics, and normal urine sodium. Hepatorenal syndrome was also less likely, given the absence of hepatic encephalopathy and normalization of synthetic function. The etiology of acute renal failure was likely APAP toxicity, leading to tubular injury.

We planned to trend his creatinine and monitor for evidence of uremia or electrolyte imbalance, either of which would necessitate emergent hemodialysis. On Day 9, despite normalization of LFTs, the patient exhibited confusion and asterixis, consistent with uremia. 

He underwent two hemodialysis sessions on Days 10 and 11. His creatinine improved afterward and continued to improve. His creatinine on discharge was 1.32 mg/dL (normal: 0.7 - 1.3 mg/dL).

## Discussion

APAP overdose can cause significant hepatic and renal toxicity. However, there have been a few case reports of AKI secondary to APAP overdose requiring hemodialysis [1-5]. Ninety percent of ingested APAP is metabolized to nontoxic compounds that are excreted unchanged in the urine. Five percent of the rest is excreted unchanged in the urine and the remainder is converted to a toxic compound, N-acetyl-p-benzoquinone imine (NAPQI), that is conjugated by hepatic glutathione and the conjugate is subsequently excreted in the urine. When APAP is ingested in a toxic amount, more acetaminophen is converted to NAPQI because the pathway to convert APAP into nontoxic compounds is saturated. Since there is not enough glutathione to conjugate this excessive amount of NAPQI, unconjugated toxic NAPQI accumulates the liver. Moreover, excessive amounts of APAP and NAPQI are excreted by the kidney and may contribute to kidney injury by tubular ischemia, resulting in acute tubular necrosis. The oxidative stress can lead to nephrotoxicity and decrease the amount of glutathione in the kidney [6]. It is important to rule out other causes of AKI, including nephrotoxic medications and hypovolemia. Treatment is generally supportive to avoid further kidney insult. There has not been much data about the role of hemodialysis, specifically, for renal toxicity. The literature supports utilizing hemodialysis for APAP overdose with signs of severe toxicity, including central nervous system depression. The dose of NAC should be doubled in such situations as it is also cleared by hemodialysis [7-8]. Our case report is consistent with the literature regarding hemodialysis use in cases of severe APAP toxicity to improve morbidity and mortality. Specifically, early hemodialysis reduces the risk of irreversible kidney damage and hemodialysis dependence. However, this area requires further investigation in the form of case-control studies or randomized control trials. 

## Conclusions

APAP overdose can cause acute renal toxicity by tubular ischemia. Little is known about chronic renal disease from the chronic use of APAP. The risk of renal failure is higher if there is co-ingestion of nonsteroidal anti-inflammatory medication or if the patient has underlying medical renal disease. APAP-induced hepatotoxicity usually precedes renal failure, and it is important to distinguish APAP-induced renal failure from the hepatorenal syndrome. APAP overdose can be treated supportively, although liver and kidney functions require close monitoring. Our case report bolsters the literature regarding urgent hemodialysis for patients with APAP toxicity exhibiting signs of uremia or with severe electrolyte disturbances.
